# Species-Level Comparative Metagenomic Analysis of the Bacterial Abundance of the Gut Microbiome in Psoriasis, Hidradenitis Suppurativa, and Pemphigus Foliaceous Patients Using Shotgun Next-Generation Sequencing

**DOI:** 10.3390/ijms27020838

**Published:** 2026-01-14

**Authors:** Lana Sá, Eleuza Machado, Verônica Ginani, Renata Timbó, Ricardo Romiti, Patrícia Kurizky, Ciro Gomes

**Affiliations:** 1 Programa de Pós-Graduação em Patologia Molecular, Faculdade de Medicina, Universidade de Brasília (UnB), Brasília 70910-900, DF, Brazil; lannaah.c@gmail.com; 2 Programa de Pós-Graduação em Ciências Médicas, Faculdade de Medicina, Universidade de Brasília (UnB), Brasília 70910-900, DF, Brazil; eleuzarodriguesmachado498@gmail.com (E.M.); renatatimbo@unb.br (R.T.); patyshu79@gmail.com (P.K.); 3 Programa de Pós-Graduação em Nutrição Humana, Faculdade de Ciências da Saúde, Universidade de Brasília (UnB), Brasília 70910-900, DF, Brazil; vcginani@unb.br; 4 Department of Dermatology, Universidade de São Paulo, São Paulo 05508-220, SP, Brazil; rromiti@hotmail.com

**Keywords:** psoriasis, hidradenitis suppurativa, immunobullous disease, microbiology

## Abstract

Recent studies have revealed a specific relationship between gut bacteria and inflammatory skin profiles. We aimed to perform a species-level comparative metagenomic analysis of the gut microbiome in patients with psoriasis, hidradenitis suppurativa (HS), and pemphigus foliaceus (PF). We included omnivorous nonsmokers and nondrinkers with psoriasis (*n* = 24), HS (*n* = 10), and PF (*n* = 11), as well as healthy controls (*n* = 10). We collected faecal samples from all patients for classic parasitological analysis. Gut microbiome analysis was conducted using shotgun next-generation sequencing. We used the Deseq2, Limma_voom, LinDA, and MaAMaAsLin 2 bioinformatics tools to evaluate concordance and differential abundance between patients. Thirteen patients (23.64%) were diagnosed with active intestinal parasitosis. The presence of intestinal parasitosis was significantly related to immunosuppression (*p* = 0.009). The most abundant microorganism species found in the faeces of the patients evaluated was *Escherichia coli*. Psoriasis patients presented a greater abundance of bacteria from the Veillonellaceae family, whereas PF patients presented a greater abundance of Firmicutes bacteria. Patients with PF showed increased *E. coli* virulence and antibiotic resistance functional markers. Immunosuppression significantly influenced the presence of intestinal parasitosis as well as increased the virulence of functional markers in patients with PF receiving systemic corticosteroid therapy.

## 1. Introduction

Immune-mediated diseases not only have substantial social and financial impacts but also, refs. [1,2], along with cardiovascular events and cancer, have shown a marked increase in incidence and prevalence over time [3]. Although these conditions have a significant genetic influence, various known and unknown environmental factors also play crucial roles [4]. The role of diet and the gut microbiota has been studied with increasing frequency, and its relationship with skin tumourigenesis and immunology appears to be highly significant [4,5,6,7,8,9]. A decrease in diversity and colonisation by specific agents has been described in the context of diseases such as psoriasis, atopic dermatitis, hidradenitis suppurativa (HS), and pemphigus [5].

Almost all studies of the intestinal microbiota in dermatological diseases have utilised 16S sequencing. Although this technique is practical and objective, it is limited by its inability to support functional assessments, its susceptibility to polymerase chain reaction biases, and its inability to identify organisms at the species level, resulting in a less detailed analysis. Shotgun sequencing, a next-generation sequencing (NGS) technique, can be used to perform more detailed functional analyses and detect all species present, including viruses, bacteria, and fungi [10,11,12,13]. Therefore, a more detailed metagenomic study of the faeces of patients with immune-mediated dermatological diseases is highly important.

We aimed to perform a species-level comparative metagenomic analysis of gut microbiome abundance in psoriasis, HS, and endemic pemphigus foliaceus (PF) patients by using shotgun NGS. We also aimed to compare the clinical characteristics and known immunopathology of each disease with our findings related to the gut microbiome to detect possible patterns associated with each disease.

## 2. Results

We included faecal samples from 55 patients. They were clinically classified as 24 patients with psoriasis, 10 patients with HS, 11 patients with PF, and 10 healthy controls. Among the psoriasis patients, 18 were taking immunosuppressive medications (methotrexate = 2; etanercept = 1; adalimumab = 7; secukinumab = 3; ustekinumab = 4; prednisone = 1). Among the patients with hidradenitis, 5 were using immunosuppressants (methotrexate = 1; mycophenolate mofetil = 1; prednisone = 2; adalimumab = 1). Finally, among the patients with PF, 10 were on immunosuppressive medications (mycophenolate mofetil + prednisone = 4; prednisone = 6).

### 2.1. Parasitological Analysis of Faecal Samples

Parasitological analysis of the faecal samples revealed that thirteen patients (23.64%) had pathological results and were referred for clinical treatment. The majority, ten patients, tested positive for *Giardia*, with *Giardia duodenalis* identified in two patients. Three patients had helminth ova in their faeces, with one case successfully identified as *Ascaris lumbricoides*. Among the patients with pathological faecal results, five had psoriasis, two had HS, and six had PF. Only one of these patients with positive faecal results was not taking immunosuppressive medication. The occurrence of diagnosed intestinal parasitosis was significantly related to the presence of immunosuppression (*p* = 0.009). Neither sex (*p* = 0.076), age (*p* = 0.956), nor disease type (*p* > 0.050) was associated with the occurrence of intestinal parasitosis. Neither the metagenomic species abundance analysis nor the functional analysis identified any differences between patients diagnosed with active intestinal parasitosis and those without a diagnosis of active intestinal parasitosis.

### 2.2. Descriptive Metagenomic Analysis

**Figure 1:**
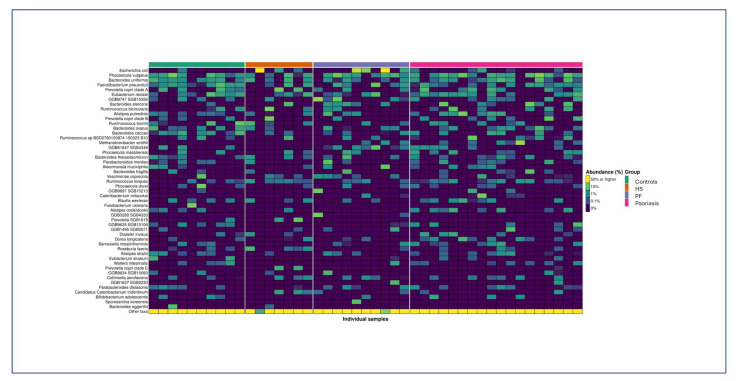
Heatmap showing the abundance of the 50 most abundant species found in the faeces of individuals from each clinical group. For a descriptive analysis of more species, please consult the supporting information. Legend: HS = hidradenitis suppurativa; PF = endemic pemphigus foliaceus.

Considering the total population, the most abundant microorganism species found in the faeces of the patients evaluated was *Escherichia coli*, followed by *Phocaeicola vulgatus*, *Bacteroides uniformis*, *Faecalibacterium prausnitzii*, and *Prevotella corpi* clade A. Figure 1 shows a heatmap as a visual representation of the abundance of the 50 most abundant species.

### 2.3. Alpha and Beta Diversity Analysis

**Figure 2:**
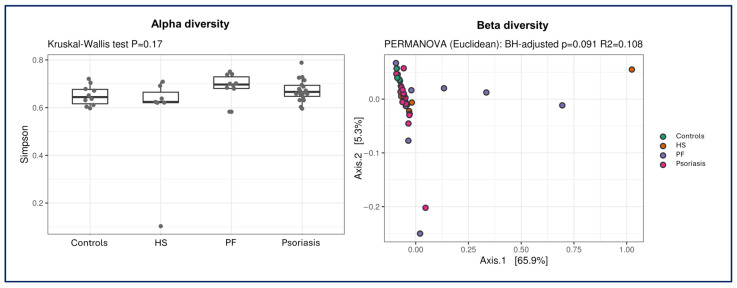
Graphs showing the distribution and analysis of alpha and beta diversity among the groups. Legend: HS = hidradenitis suppurativa; PF = endemic pemphigus foliaceus.

The alpha and beta diversity analysis models revealed no differences between the groups (Figure 2).

### 2.4. Differential Abundance Analysis (DAA)

**Figure 3:**
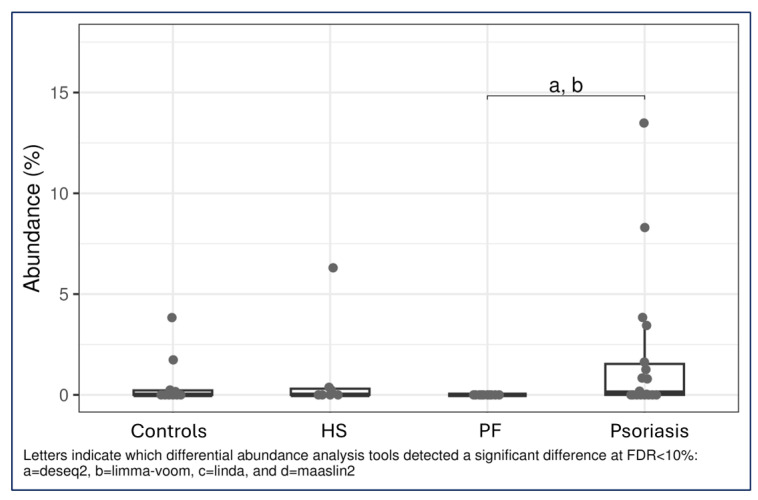
Graph showing relative abundance and concordance obtained using various bioinformatics tools. The most notable differences in abundance were observed when psoriasis patients were compared with pemphigus foliaceus patients. The results obtained with Deseq2 and Limma_voom were consistent and revealed that patients with psoriasis presented a greater abundance of bacteria from the Veillonellaceae family in their faeces than did patients with pemphigus foliaceus. Legend: HS = hidradenitis suppurativa; PF = endemic pemphigus foliaceus; FDR = false discovery rate.

**Figure 4:**
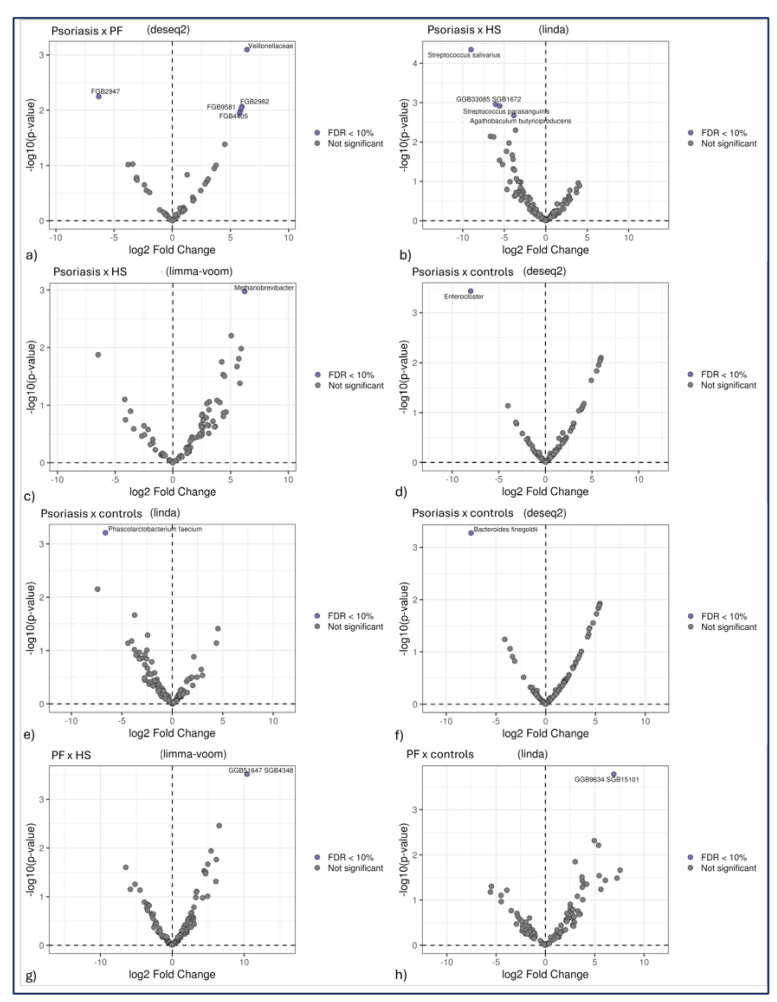
(**a**–**h**) Graphs showing relative abundance and significant differences, as identified with different bioinformatics tools. Legend: HS = hidradenitis suppurativa; PF = endemic pemphigus foliaceus; FDR = false discovery rate.

The most notable differences in abundance were observed when psoriasis patients were compared with PF patients. Deseq2 and Limma_voom were consistent in revealing that compared with patients with PF, patients with psoriasis presented a greater abundance of bacteria from the Veillonellaceae family in their faeces (Figure 3). Additionally, compared with PF patients, psoriasis patients presented a greater abundance of bacteria from the phylum Bacteroidetes (k__Bacteria|p__Bacteroidetes|c__CFGB4405) according to Deseq2 (Figure 4). Other bacteria from the phylum Firmicutes presented variable abundances, which were not significant, when psoriasis patients and PF patients were compared.

We detected a lower abundance of bacteria from the family Streptococcaceae (linear models for differential abundance analysis of microbiome compositional data (LinDa)), specifically the genus *Streptococcus* (LinDa), in the faeces of psoriasis patients than in those of HS patients. This disparity was due to a reduced abundance of the species *Streptococcus parasanguinis* and *Streptococcus salivarius* (LinDa) in psoriasis patients (Figure 4). Furthermore, compared with HS patients, a lower abundance of the genus *Agathobaculum* (Linda), represented by the species *Agathobaculum butyriciproducens* (LinDa), was detected in psoriasis patients (Figure 4). Conversely, we also detected a greater prevalence of the genus *Methanobrevibacter* in the faeces of psoriasis patients than in those of HS patients (Limma_voom) (Figure 4).

The genus *Enterocloster* (Deseq2) and the species *Phascolarctobacterium faecium* (LinDA) and *Bacteroides finegoldii* (Deseq2) were significantly less abundant in the faeces of psoriasis patients than in those of healthy controls. Compared with HS patients and healthy controls, PF patients presented a greater abundance of bacteria from the phylum Firmicutes (k__Bacteria|p__Firmicutes|c__CFGB4806|o__OFGB4806|f__FGB4806|g__GGB51647|s__GGB51647_SGB4348|t__SGB4348 (Limma_voom) and k__Bacteria|p__Firmicutes|c__Clostridia|o__Eubacteriales|f__Oscillospiraceae|g__GGB9634|s__GGB9634_SGB15101 (Linda)) (Figure 4). No significant difference was found when comparing HS patients to controls.

### 2.5. Functional Analysis

**Figure 5:**
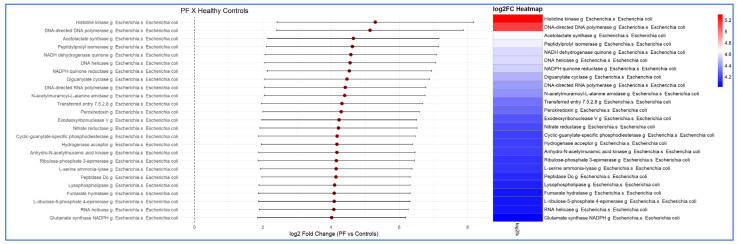
Presentation of the 25 most significantly upregulated metabolic signatures, in descending order, identified in the functional analysis of the intestinal microbiota of patients with pemphigus foliaceus (PF) compared with healthy controls. The results indicate increased metabolic activity derived from *Escherichia coli* in PF patients relative to healthy controls. Differences in expression are presented as log2 fold change (FC) (LinDa).

**Figure 6:**
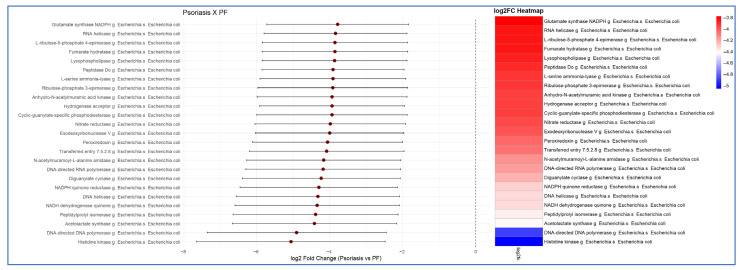
Presentation of the 25 most significantly upregulated metabolic signatures, in descending order, identified in the functional analysis of the intestinal microbiota of patients with psoriasis compared with pemphigus foliaceus (PF) patients. The results indicate increased metabolic activity derived from *Escherichia coli* in PF patients relative to that in psoriasis patients. Differences in expression are presented as −log2 fold change (FC) (LinDa).

The functional analysis consistently demonstrated, with at least two concordant bioinformatics models, a higher metabolic activity of the intestinal microbiota in patients with PF compared with those in patients with psoriasis and healthy controls. This increased activity was clearly identified using the Enzyme Commission number (EC) and MetaCyC database classifications. The 25 most significant functional signatures, exhibiting the greatest fold-change in the comparison between PF patients and healthy controls, are presented in Figure 5. The comparison between the psoriasis and PF patient groups is shown in Figure 6.

Markers of increased virulence were also more abundant in the functional metagenomic analysis of the faeces of PF patients compared with psoriasis patients and healthy controls. Among the most notable virulence-associated markers related to *E. coli* and found in higher abundance were beta-galactosidase (EC 3.2.1.23), glutathione-specific gamma-glutamylcyclotransferase (EC 4.3.2.7), shikimate dehydrogenase (EC 1.1.1.25), various enzymes of the phosphotransferase system (e.g., protein-Npi-phosphohistidine-D-mannose phosphotransferase, EC 2.7.1.191), and fimbrial assembly-associated enzymes (e.g., tRNA pseudouridine synthases, EC 5.4.99.12 and EC 5.4.99.25).

**Figure 7:**
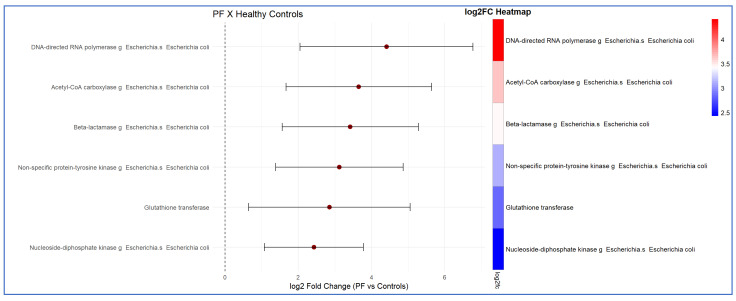
Presentation of the *Escherichia coli* antibiotic resistance markers, in descending order, identified in the functional analysis of the intestinal microbiota of patients with pemphigus foliaceus (PF) compared with healthy controls. The results indicate increased metabolic activity of those markers in PF patients relative to healthy controls. Differences in expression are presented as log2 fold change (FC) (LinDa).

**Figure 8:**
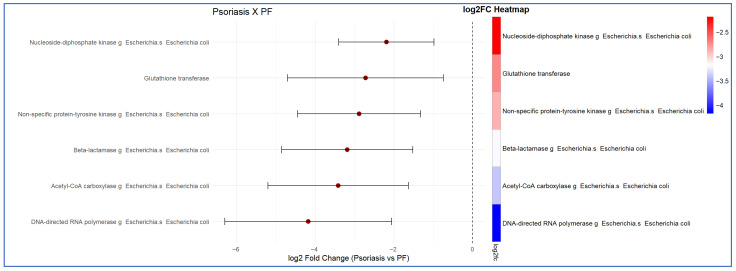
Presentation of the *Escherichia coli* antibiotic non-response markers, in descending order, identified in the functional analysis of the intestinal microbiota of patients with psoriasis compared with pemphigus foliaceus (PF) patients. The results indicate increased metabolic activity derived from *Escherichia coli* in PF patients relative to psoriasis patients. Differences in expression are presented as −log2 fold change (FC) (LinDa).

Compared with psoriasis patients and healthy controls, other notably important markers that were found in greater abundance in the faecal analysis of PF patients were antimicrobial resistance markers. The function of each of these selected antimicrobial resistance markers is detailed in Table 1. The fold changes observed in the comparison between PF patients and healthy controls, as well as between psoriasis and PF patients, with respect to antimicrobial resistance markers, are presented in detail in Figure 7 and Figure 8, respectively.

## 3. Discussion

The interaction between the skin and the gut microbiome is a topic of intense discussion. Although there is no doubt that an altered gut microbiota is directly related to inflammatory skin diseases, the details of this association are still being elucidated. This study involved a comprehensive and detailed analysis of the gut microbiome in patients with psoriasis and HS. We also included a group of patients with endemic PF, a form of PF that is common in Brazil but not commonly present in some regions of the world. Before inclusion, all patients were matched according to clinical characteristics such as disease severity, sex, and age. More severe forms of disease are characterised by greater disruption of immunological physiology, making any systemic pattern, including those of the microbiota, more evident.

Starting with a classic parasitological analysis, we found that thirteen patients (23.64%) had pathological results. Among these patients, ten were diagnosed with giardiasis. Giardiasis is a cosmopolitan disease and an important cause of waterborne and foodborne diarrhoea [19]. Another three patients were diagnosed with helminthiasis, also known as cosmopolitan intestinal parasitosis [20]. Our study revealed that immunosuppression was significantly associated with a diagnosis of intestinal parasitosis. These results highlight the necessity of constant vigilance for this type of infection in any patient with immune-mediated diseases to avoid undesirable or severe symptoms related to growing infestations. The presence of active intestinal parasitosis in patients could be a significant confounding factor in metagenomic analyses [21,22] of both abundance and function. However, we did not observe any statistically significant differences in these analyses when comparing subgroups with or without intestinal parasitosis.

The metagenomic analysis of the faecal samples yielded interesting results. Although new data on endemic PF, a region-specific disease in Brazil, are particularly noteworthy, our findings on the gut microbiome in psoriasis patients also validated those of previous studies. The most abundant microorganism species in all the groups was *E. coli*. *E. coli* is considered the predominant bacterium within the Enterobacteriaceae family in the healthy human gut [23,24]. Debate regarding the role of *E. coli* in inflammation is ongoing, but some studies have reported enrichment of this bacterium in patients with inflammatory bowel diseases and even psoriasis [24,25,26]. Some subtypes of this bacterium are suspected to be more pathogenic and act invasively or by producing toxins [25]. Alpha and beta diversity analyses did not reveal differences between the groups. This result may indicate successful matching, meaning that the sample was homogeneous after the matching techniques were applied. Environmental factors, including dietary habits, are likely more significant in defining the intestinal microbiota than are the studied diseases, which may influence but perhaps not radically change the intestinal microbiota. Possible factors such as successful matching, the predominant influence of a shared environment or diet, or insufficient statistical power may account for the absence of significant differences in alpha and beta diversity.

The family Veillonellaceae belongs to the phylum Firmicutes and the class Negativicutes [27]. Members of the Veillonellaceae family are obligate anaerobes that are typically found in aquatic environments and the intestines of vertebrates and can rarely cause opportunistic infections in humans [27]. Previous studies have shown an increased abundance of members of the Veillonellaceae family in oral, stool, and intestinal tissue samples from patients with Crohn’s disease [28]. A relatively high abundance of bacteria from this family in psoriasis patients has also been previously reported, suggesting a phenotypic link between inflammatory bowel diseases and psoriasis [29,30]. However, how this specific faecal microbiota pattern is related to the occurrence or severity of psoriasis remains unclear [29,30]. In our study, an analysis of the Veillonella family revealed that members of this family were less abundant in PF patients than in those with psoriasis. This finding appears to be quite consistent with reports in the literature. Previous studies have shown that the abundance of the Veillonella family is negatively related to pathogenic antibodies in patients with pemphigus vulgaris [31]. However, whether this pattern is specific to pemphigus diseases or can be expected in diseases predominantly mediated by antibodies produced by B lymphocytes is unknown.

The relative abundances of the phyla Firmicutes and Bacteroidetes have been widely studied, as they are the most prevalent bacteria in the human gut microbiota. This study revealed an increased abundance of Bacteroidetes in patients with psoriasis compared with those with PF. In some studies that compared psoriasis and HS patients with healthy controls, the abundance of the phylum Bacteroidetes increased, whereas the abundance of Firmicutes decreased [24,32]. Other studies also reported the opposite results, showing that the Firmicutes/Bacteroidetes ratio is elevated in psoriasis patients [33].

Regardless of the direction of change, as numerous environmental, geographical, dietary, and clinical factors influence this relationship, any disturbance in the Firmicutes/Bacteroidetes ratio may be associated with systemic inflammation [34]. Dysbiosis, or an imbalance between these bacterial groups, can contribute to conditions such as obesity, inflammatory bowel disease, and metabolic syndrome [35]. In the present study, we included only severe and active cases of PF, and faecal samples from this group of patients presented a greater abundance of Firmicutes than those from HS patients and healthy controls did. Previous data revealed that the Firmicutes/Bacteroidetes ratio tended to decrease in patients in the remission phase of pemphigus vulgaris [31].

We detected a lower abundance of bacteria from the genus *Streptococcus* in the faeces of psoriasis patients than in those of HS patients. Similarly, *Streptococcus* spp. was more abundant in HS patients than in healthy controls [6]. The presence of a greater quantity of *Streptococcus* in the faeces is related to an unknown association with HS, but this finding in the faeces has been reported to have a positive association with inflammatory markers [36]. We also detected a greater prevalence of the genus *Methanobrevibacter* in the faeces of psoriasis patients than in those of HS patients. Previous studies have suggested that *Methanobrevibacter* is linked to psoriatic arthritis. *Methanobrevibacter* is not only a marker of inflammation and intestinal barrier dysfunction but also linked to a shift towards Th17-mediated inflammation, involving enhanced activity of the interleukin-17 and interleukin-23 pathways, which are crucial immunological drivers. This association is likewise significant in the context of psoriatic arthritis [37].

Functional analysis revealed a consistent enrichment of Enterobacteriaceae-associated metabolic pathways in patients with PF, an alteration absent in psoriasis, HS, and healthy control groups. This disease-specific shift in gut microbial ecology appears intrinsically linked to the systemic inflammation and immune dysregulation characteristic of PF, as well as the frequent administration of immunosuppressive therapies. Patients with autoimmune blistering diseases still rely heavily on systemic oral corticosteroid therapy [38]. Oral corticosteroids are broad-spectrum immunosuppressants associated with a higher rate of adverse events and immunosuppression compared with the targeted therapies currently approved for psoriasis [39]. Such factors may disrupt the homeostasis of the intestinal microbiome, thereby favouring the proliferation of facultative anaerobes such as Enterobacteriaceae [40].

Our functional metagenomic analysis further demonstrated that PF patients displayed marked enrichment of *E coli*-associated metabolic signatures. These encompassed pathways involved in DNA replication and repair, energy metabolism under inflammatory conditions, cell wall remodelling, and regulation of stress responses [41]. Additionally, the upregulation of peptidoglycan turnover enzymes, peroxiredoxins, and stress-induced proteases suggests that *E. coli* populations in PF patients are actively adapting to oxidative and immune-mediated stress [42].

Notably and of particular interest, the enrichment of *E. coli*-derived virulence markers and markers indicative of antibiotic resistance [14,15,16,17,18] may signal an increased risk of complications in patients with PF, especially those frequently receiving high doses of oral corticosteroids [43,44]. *E. coli* is associated with the development of gastroenteritis and urinary tract infections, and these complications warrant vigilant monitoring by the clinical care team in affected patients [43]. In contrast, psoriasis, despite its inflammatory character, generally maintains a more resilient intestinal barrier and effective antimicrobial defences, thereby limiting the expansion of Enterobacteriaceae. No evidence exists of a higher risk of severe gastrointestinal and urinary infectious complications in psoriasis, a phenomenon widely reported in cohorts of patients receiving biologic therapies [45].

In the present study, the principal limitations include the limited sample size and the potential confounding effects of the diverse treatment regimens administered to participants. As metagenomic analysis is an advanced molecular technique, sample sizes in the literature are typically restricted; however, the ongoing publication of such studies may facilitate future pooled analyses or meta-analyses. Furthermore, controlling for the majority of variables in observational studies is not feasible, with heterogeneity in treatment among patients with matched disease activity representing an additional variable. Nonetheless, this analysis remains valuable, particularly because contemporary treatments for psoriasis employ targeted agents rather than broad-spectrum immunosuppressants, in contrast to PF, which still relies predominantly on systemic corticosteroids with potent immunosuppressive effects.

## 4. Materials and Methods

### 4.1. Patients and Settings

We recruited a convenience sample comprising patients with psoriasis, PF, and HS and healthy controls. Since there is a greater frequency of psoriasis samples reported in the literature, we predicted a 2:1 ratio in terms of the number of patients with psoriasis compared with the number of patients in the other groups. We predicted that at least 20 patients with psoriasis, 10 patients with HS, 10 patients with PF, and 10 healthy controls would be included. Clinical homogenisation of the patients was conducted before inclusion in the study, with all patients being omnivores, nonsmokers, and nondrinkers and having not reported dietary changes (diets, dietary restrictions, or other changes) or changes in residence in the previous six months. Patients could not have other underlying illnesses, such as chronic infections, hypertension, depression, anxiety disorders, diabetes mellitus, or any other acute or chronic pathology, at the time of sample collection. Patients in the different groups were matched in terms of age and sex (Fisher’s exact test: *p* > 0.100). Only patients with severe forms of the respective pathologies were included; severe psoriasis was indicated by a psoriasis area and severity index (PASI) > 10, severe HS was indicated by a Hurley stage III classification, and severe PF was indicated by body lesions covering more than 10% of the skin surface. Treatment regimens were neither used as matching criteria nor considered as grounds for exclusion from the study, as we included only patients with uncontrolled active disease. Furthermore, enrolling severely affected, untreated patients presents considerable ethical and technical challenges. Even patients undergoing systemic treatment had active disease: in psoriasis, a PASI > 10; in HS, an International Hidradenitis Suppurativa Severity Scoring System ≥ 11; and in PF, more than 10% of the body affected by active blistering lesions.

### 4.2. Parasitological Analysis of Faecal Samples

The samples were collected using a plastic spatula in 80 mL universal collection containers (JPROLAB, São José dos Pinhais, Brazil), sterilised by ionising radiation without preservatives. At the time of delivery of the collection containers, the participants received instructions orally and in writing on how to collect the stool sample, including a request to keep the samples refrigerated until delivery. The containers containing the samples were collected from the participants’ homes, packed in a Styrofoam box with a lid, cooled with 200 mL (2 × 7 × 2.9 cm) reusable rigid artificial ice gel plates (GELOTECH, Pinhais, Brazil), and transported to the Dermatology Laboratory at the University of Brasília.

For the parasitological examination, the samples were processed using the spontaneous sedimentation method. The samples were kept in the refrigerator (4 °C) until evaluation. Three researchers prepared and evaluated 15 slides per sample. To prepare the slides, we placed a drop of the processed sample on a microscope slide, added a drop of Lugol’s stain (5% iodine and potassium iodide solution), and covered the slide with a microscope cover slip. The slides were viewed under an optical microscope BX41 (Olympus Corporation, Tokyo, Japan) at magnifications ranging from 100× to 1000× (10× to 100× objectives), and the entire field delimited by the cover slip (24 × 40 mm) was included, with a time limit of 5 min per slide.

### 4.3. Methodology of Intestinal Microbiome Analysis—Metagenomics

A sample of 2 g of total faeces from each patient was stored at −80 °C and subsequently transported on dry ice to a specialised company (BIOMEHUB, Florianópolis, Brazil), where the metagenomic analysis of the samples was conducted.

### 4.4. DNA Extraction, Library Preparation, and Sequencing

DNA extraction was performed using the ZymoBIOMICS DNA Miniprep Kit (Zymo Research, Irvine, CA, USA). The analysis of the microbial composition of the samples was carried out by metagenomics using random enzymatic digestion of DNA with the Illumina DNA Prep Kit (Illumina Inc., San Diego, CA, USA). The final DNA concentration of the library set was estimated using Picogreen (Invitrogen, Waltham, MA, USA) and then precisely quantified by real-time PCR using the Collibri Library Quantification Kit (Invitrogen, Waltham, MA, USA). After the quantification step, the samples were sequenced on a NextSeq 1000 instrument (Illumina Inc., San Diego, CA, USA) using the standard Illumina primers provided by the manufacturer’s kit. Paired-end sequencing (150 × 150 bp) was performed with the NextSeq 1000/2000 P1 Reagents Kit (Illumina, San Diego, CA, USA), which produced an average of 1 million reads/sample.

### 4.5. Bioinformatics Analyses

The sequences obtained were subjected to a pipeline analysis developed by BiomeHub (v1, Florianópolis, Brazil) for metagenome analysis. The analysis involves quality control of the raw fastq files using the Biobakery Kneaddata pipeline (https://huttenhower.sph.harvard.edu/kneaddata/, accessed on 16 July 2024) with sequencing adapter sequences and low-quality bases removed using Trimmomatic v.0.33 [46]. Decontamination was performed using Bowtie2 (v2.2) [47] with the human genome (NCBI accession number: GCF_000001405.26). Additionally, quality files compiled in fastqc (v0.11.9) (https://www.bioinformatics.babraham.ac.uk/projects/fastqc/, accessed on 16 July 2024) were provided. Taxonomic identification was performed using Metaphlan 4.0.6 (https://doi.org/10.1101/2022.08.22.504593), which utilises the CHOCOPhlAnSGB version 202212 database; this database contains approximately 5.1 M unique clade-specific gene markers identified from ~1 M microorganism genomes (https://huttenhower.sph.harvard.edu/metaphlan/, accessed on 16 July 2024). The taxonomic profile results of each sample were analysed together, in terms of absolute count and relative abundance, using BiomeHub encodestats software (v1.0.14).

Gene family prediction was performed using the HUMAnN 3 software (v3.0+) [48] with the uniref90 database [49], taking into account the taxonomic classification provided by Metaphlan. In this manner, gene families were predicted and stratified by taxonomy. Subsequently, these families were regrouped and quantified into: metabolic pathways according to the classifications in the MetaCyC database; Ref [50] molecular functions based on the KEGG orthologues database; ontological annotations, ref [51] utilising the Gene Ontology [52] and EggNOG [53] databases; enzymatic families following the Enzyme Commission number (EC); and protein families according to the PFAM database [54].

The taxonomic profile results for each sample, along with the functional data resulting from classification across the various databases, were analysed collectively, considering both absolute count (number of reads) and relative abundance, using the BiomeHub encodestats software. In this manner, results for alpha and beta diversity, descriptive analysis, and differential abundance analysis were obtained, with comparisons established according to the sample groupings specified by the client in the metadata. The functional results from MetaCyC were presented both in full and by their reaction subgroups (MetaCyC reactions-rxn). Taxonomic and functional comparisons were performed between the different dermatological groups included in the study, as well as according to the presence or absence of active intestinal parasitosis. Group homogeneity for other characteristics was sought by matching the populations at the time of inclusion. In this paper, the functional results were expressed according to the EC classification in order to generate a standardized, universally recognized classification and a better relationship with clinical phenotypes. For the analysis of additional types of data, please consult the supporting information. All individual results from the functional analyses underwent a thorough review of the literature, aiming to identify markers of virulence and antibiotic resistance.

### 4.6. Statistical Analysis

We discerned patterns in the distributions of the top 10, 50, 100, and 400 most prevalent species. Alpha-diversity analyses revealed the variety of species within individuals, whereas beta-diversity analyses evaluated the differences between groups. Following these initial steps, we evaluated the quantitative abundance of the gut microbiota across different groups, employing a comprehensive strategy for differential abundance analysis (DAA).

All statistical analyses were conducted using R software (v. 4.3.1) (Posit Team, Boston, MA, USA), employing the phyloseq (v. 1.44.0) [55] and tidyverse (v. 2.0.0) [56] packages. Although certain analyses are more commonly reported at specific taxonomic levels (for instance, alpha and beta diversity are typically assessed using oligotype/ASV data), each analysis described was independently performed across all the following taxonomic levels: oligotype/ASV, lowest (the lowest taxonomic level classified for each ASV), species, genus, family, and phylum.

### 4.7. Alpha Diversity

Alpha diversity analysis was used to determine the abundance of all species within all patient samples in each group. The data were visualised as box plots, and the groups were compared using the Kruskal–Wallis test. The following metrics were also visualised: the Shannon, Simpson, Inverse Simpson, and Richness indices.

### 4.8. Beta Diversity Analysis

Beta diversity analysis was used to evaluate the differences in species composition between the psoriasis patients, HS patients, PF patients, and controls. Nonmetric multidimensional scaling and principal coordinate analysis plots were used to visualise the beta diversity results. For the analysis, we compared the Bray–Curtis, Jaccard, and Euclidean distances via permutational multivariate analysis of variance (PERMANOVA).

### 4.9. Differential Abundance Analysis (DAA)

Since changes in the composition of the gut microbiota can be spurious and depend on various environmental factors [57], we used different bioinformatics methods to evaluate the differential abundance between patients. Consensus between these methods was evaluated.

Deseq2: Deseq2 is a conservative approach that can be used to infer metagenomic gene count data by assuming a negative binomial distribution for count distribution [58].

Limma_voom: This technique estimates the relationship between the mean and variance of log-counts, assigns a precision weight to each observation, and integrates these weights into the limma empirical Bayes analysis pipeline. The limma package (v3.28.14), which was originally designed for differential expression analysis of microarray data, has been adapted with the voom function to handle RNA-Seq data [59]. For the functional analyses, since the data were normalised, the limma_trend method was employed.

LinDA: Linear models for differential abundance analysis of microbiome compositional data (LinDA) is regarded as a simplified yet conservative method for microbiome analysis. It fits linear regression models on centred log-ratio-transformed data while correcting for biases caused by compositional effects [60].

MaAsLin 2: Microbiome multivariable associations with linear models (MaAsLin 2) employs linear and mixed models to suit a wide range of contemporary epidemiological studies. This includes studies with both cross-sectional and longitudinal designs, accommodating various data types such as count data and relative abundance data [61].

Statistical significance was set with a *p* value < 0.05 and a 95% confidence interval (CI). False discovery rate (FDR) adjustments were made for all analyses of differential abundance. Statistical analysis was performed with the survival and survminer packages in R version 4.4.2 (R Core Team (2021); R: A language and environment for statistical computing; R Foundation for Statistical Computing, Vienna, Austria; URL https://www.R-project.org/).

### 4.10. Ethics Statement

The procedures adopted followed the Declaration of Helsinki. The study and all research procedures adopted were approved by the Research Ethics Committee of the Faculty of Medicine of the University of Brasília (47377121.7.1001.5558).

## 5. Conclusions

Our study reveals significant metagenomic differences in the gut microbiome among patients with immune-mediated skin diseases and underscores the importance of traditional parasitological exams. This finding necessitates ongoing vigilance and early intervention for these infections.

## Figures and Tables

**Table 1:** List of *Escherichia coli* antibiotic resistance markers that were found to be more abundant in patients with pemphigus foliaceus (PF) than in the intestinal microbiota analysis of patients with psoriasis and healthy controls.

Gene Signatures Derived from EC Numbers	Antibiotic Resistance Markers
Beta-lactamase (EC 3.5.2.6)	Primary marker for beta-lactam antibiotic non-response in *E. coli* [14].
Acetyl-CoA carboxylase (EC 6.4.1.2)	Can reduce aminoglycoside uptake, supporting resistance phenotypes [15].
DNA-directed RNA polymerase (EC 2.7.7.6)	Mutations affecting this enzyme can confer rifampicin resistance [16].
Non-specific protein-tyrosine kinase (EC 2.7.10.2), nucleoside-diphosphate kinase (EC 2.7.4.6)	Implicated in antibiotic efflux and biofilm formation, contributing to antibiotic tolerance [17].
Glutathione transferase (EC 2.5.1.18)	Overexpression is associated with multidrug resistance by reducing intracellular drug concentrations [18].

Legend: EC = Enzyme Commission number.

## Data Availability

All the data generated in this study can be accessed via Mendeley data: Gomes, Ciro; Sá, Lana (2025), “Species-level comparative metagenomic analysis of gut microbiome bacterial abundance in psoriasis, hidradenitis suppurativa, and pemphigus foliaceous patients using shotgun next-generation sequencing”, Mendeley Data, V1, https://doi.org/10.17632/yjz7v8rknx.1 and V2, https://doi.org/10.17632/yjz7v8rknx.2.

## Abbreviations

The following abbreviations are used in this manuscript:

HS	Hidradenitis suppurativa
PF	Pemphigus foliaceus
NGS	Next-generation sequencing
EC	Enzyme Commission number
PERMANOVA	Permutational multivariate analysis of variance
DAA	Differential abundance analysis
PASI	Psoriasis area and severity index
LinDA	Linear models for differential abundance analysis of microbiome compositional data
MaAsLin 2	Microbiome multivariable associations with linear models
FDR	False discovery rate
